# M-test in linear models with negatively superadditive dependent errors

**DOI:** 10.1186/s13660-017-1509-6

**Published:** 2017-09-22

**Authors:** Yuncai Yu, Hongchang Hu, Ling Liu, Shouyou Huang

**Affiliations:** 1 State Key Laboratory of Mechanics and Control of Mechanical Structures, Institute of Nano Science and Department of Mathematics, Nanjing University of Aeronautics and Astronautics, Nanjing, 210016 China; 2 Department of Mathematics and Statistics, Hubei Normal University, Huangshi, 435002 China; 3 Department of Information Science and Technology, Donghua University, Shanghai, 201600 China

**Keywords:** 60F05, 60G10, 62F35, 62M10, 60G42, NSD random sequences, linear regression models, M-test, asymptotic property, Monte Carlo simulations

## Abstract

This paper is concerned with the testing hypotheses of regression parameters in linear models in which errors are negatively superadditive dependent (NSD). A robust M-test base on M-criterion is proposed. The asymptotic distribution of the test statistic is obtained and the consistent estimates of the redundancy parameters involved in the asymptotic distribution are established. Finally, some Monte Carlo simulations are given to substantiate the stability of the parameter estimates and the power of the test, for various choices of M-methods, explanatory variables and different sample sizes.

## Introduction

Consider the linear regression model:

1 $$ y_{t}= \mathbf{x}_{t}^{\top} \boldsymbol { \beta} +e_{t}, \quad t=1,\ldots,n, $$

where $\{y_{t}\}$ and $\{\mathbf{x}_{t}=(x_{t1},x_{t2},\ldots ,x_{tp})^{\top}\}$ are real-valued responses and real-valued random vectors, respectively. The superscript ⊤ represents the transpose throughout this paper, $\boldsymbol {\beta}=(\beta_{1},\ldots,\beta _{p})^{\top}$ is a *p*-vector of the unknown parameter, and $\{e_{t}\}$ are random errors.

It is well known that linear regression models have received much attentions for their immense applications in various areas such as engineering technology, economics and social sciences. Unfortunately, there exists the problem that the classical maximum likelihood estimator for these models is sufficiently sensitive to outliers. To overcome this defect, Huber proposed the M-estimate which possesses the robustness (see Huber [1]) by minimizing

2 $$ \sum_{t=1}^{n}\rho \bigl(y_{t}- \mathbf{x}_{t}^{\top} \boldsymbol {\beta} \bigr), $$

where *ρ* is a convex function. It is obvious that many important estimates can be addressed easily. For instance, the least square (LS) estimate with $\rho(x)={x^{2}}/{2}$, the least absolute deviation (LAD) estimate with $\rho(x)=|x|$, and the Huber estimate with $\rho (x)={(x^{2}I(|x|\leq k))}/2+(k|x|-{k^{2}}/2)I(|x|>k)$, $k>0$, where $I(A)$is the indicator function of *A*.

Let $\hat{\beta}_{n}$ be a minimizer of (2) and consequently $\hat {\beta }_{n}$ is a M-estimate of *β*. Some excellent results as regards the asymptotic properties of $\hat{\beta}_{n}$ with various forms of *ρ* have been reported in [2–5]. Most of the results rely on the independence errors. As Huber claimed in [1], the independence assumption on the errors is a serious restriction. It is practically essential and imperative to explore the case of dependent errors, which is a theoretically challenging. Under the dependence assumption of the errors, Berlinet *et al.* [6] proved the consistency of M-estimates for linear models with strong mixing errors. Cui *et al.* [7] obtained the asymptotic distributions of M-estimates for linear models with spatially correlated errors. Wu [8] investigated the weak and strong Bahadur representations of the M-estimates for linear models with stationary causal errors. Wu [9] established the strong consistency of M-estimates for linear models with negatively dependent (NA) random errors.

In the following we will introduce a wide random sequence, NSD random sequence, whose definition based on the superadditive functions.

### Definition 1

Hu [10]

A function $\phi: \mathbb{R}^{n}\rightarrow\mathbb{R}$, is called superadditive if

$$\phi( \mathbf{x}\vee\mathbf{y})+\phi( \mathbf {x}\wedge\mathbf{y})\geq\phi( \mathbf{x})+\phi( \mathbf{y}), $$

for all $\mathbf{x}, \mathbf{y}\in\mathbb{R}^{n}$, where ‘∨’ is for a componentwise maximum and ‘∧’ is for a componentwise minimum.

### Definition 2

Hu [10]

A random vector $(X_{1},X_{2},\ldots,X_{n})$ is said to be NSD if

3 $$ E\phi(X_{1},X_{2},\ldots,X_{n})\leq E\phi\bigl(X_{1}^{*},X_{2}^{*}, \ldots,X_{n}^{*}\bigr), $$

where $\{X_{t}^{*}, t=1,\ldots,n\}$ are independent random variables such that have same marginal distribution with $\{X_{t},t=1,\ldots,n\}$ for each *t*, and *ϕ* is a superadditive function such that the expectations in (3) exist.

### Definition 3

Wang *et al.* [11]

A sequence of random variables $(X_{1},\ldots,X_{n})$ is called NSD if for all $n\geq1$, $(X_{1},\ldots,X_{n})$ is NSD.

The concept of NSD random variables, which generalizes the concept of NA, was proposed by Hu [1]. In such paper, author provided several essential properties and valuable theorems for NSD. It is realized that many multivariate distributions possess the NSD property exhibited in practical examples. Compared with NA, NSD contains more widely sequence [12], *i.e.*, NA means NSD, but not vise verse. Consequently, NSD has received an increasing attention for its enormous research value in comparison with NA both in copula theory and applications [13–19]. Specifically, a Kolmogorov and a Rosenthal inequality of NSD random variables are introduced in [16] and [17], respectively. Furthermore, Wang *et al.* [11] obtained the complete convergence for weighted sums of NSD random variables and investigated the complete consistency of LS estimates in the EV models. Wang *et al.* [19] established the strong consistency of M-estimates for linear models with NSD errors via improving the moment condition.

The purpose of this paper is to investigate the M-test problem of the regression parameters in the model (1) with NSD random errors, we consider a test for the following hypothesis:

4 $$ H_{0}: \mathbf{H}^{\top}(\boldsymbol {\beta} - \mathbf{b}) = \mathbf{0} \quad \text{versus} \quad H_{1}: \mathbf{H}^{\top }(\boldsymbol {\beta} - \mathbf{b}) \neq\mathbf{0}, $$

where **H** is a known $p\times q$ matrix with the rank *q* $(0< q\leq p)$, **b** is a known *p*-vector.

A sequence of the local alternatives is considered as follows:

5 $$ H_{2,n}: \mathbf{H}^{\top}(\boldsymbol {\beta} - \mathbf {b}) = \mathbf{H}^{\top}\boldsymbol {\omega}_{n}, $$

where $\boldsymbol {\omega}_{n}$ is a known *p*-vector such that

6 $$ \bigl\Vert \mathbf{S}_{n}^{1/2}\boldsymbol { \omega}_{n} \bigr\Vert =O(1), $$

where $\mathbf{S}_{n}=\sum_{t=1}^{n}\mathbf {x}_{t}\mathbf{x}_{t}^{T}$, $\|\boldsymbol {\cdot}\|$ is the Euclidean norm.

Denote

$$\begin{aligned}& \min_{\mathbf{H}^{\top}(\boldsymbol {\beta} - \mathbf {b})=\mathbf{0}}\sum_{t=1}^{n} \rho\bigl(y_{t}-\mathbf{x}_{t}^{\top}\boldsymbol {\beta} \bigr)=\sum_{t=1}^{n} \rho \bigl(y_{t}-\mathbf{x}_{t}^{\top}\tilde{\boldsymbol { \beta}}\bigr), \\& \min_{\boldsymbol {\beta} \in\mathbb{R}^{p}}\sum_{t=1}^{n} \rho\bigl(y_{t}-\mathbf{x}_{t}^{\top}{\boldsymbol {\beta}} \bigr)=\sum_{t=1}^{n} \rho \bigl(y_{t}-\mathbf{x}_{t}^{\top}\hat{\boldsymbol {\beta}} \bigr), \\& {M_{n}}=\sum_{t=1}^{n} \rho \bigl(y_{t}-\mathbf{x}_{t}^{\top}\tilde{\boldsymbol {\beta }}\bigr)-\sum_{t=1}^{n} \rho \bigl(y_{t}-\mathbf{x}_{t}^{\top}\hat{\boldsymbol {\beta}} \bigr). \end{aligned}$$

Actually, $\tilde{\boldsymbol {\beta}}$ and $\hat{\boldsymbol {\beta}}$ are the M-estimates in the restricted and unrestricted model (1), respectively. To test the hypothesis (4), we adopt M-criterion which regards $M_{n}$ as the criterion to measure the level of departure from the null hypothesis. Several classical conclusions have been presented in [20–22] when the errors are assumed to be independence, we will generalize the case to NSD random errors. Throughout this paper, let *C* be a positive constant. Put $|\boldsymbol {\tau}|=\max_{1\leq t \leq p}\{|\boldsymbol {\tau }_{1}|,|\boldsymbol {\tau}_{2}|,\ldots,|\boldsymbol {\tau}_{p}|\}$ if ***τ*** is a *p*-vector. Let $x^{+}=xI(x\geq0)$ and $x^{-}=-xI(x< 0)$. A random sequence $\{X_{n}\}$ is said to on $L_{q}$-norm, $q>0$, if $E|X_{n}|^{q} < \infty$. Denote $a_{n}=o_{P}(b_{n})$ if $a_{n}/b_{n}$ converges to 0 in probability and $a_{n}=O_{P}(b_{n})$ if $a_{n}/b_{n}$ converges to a constant in probability.

The rest of the paper is organized as follows. In Section 2, the asymptotic distribution of $M_{n}$ is obtained with the NSD random errors, and the consistence estimates of the redundancy parameters *λ* and $\sigma^{2}$ are constructed under the local hypothesis. Section 3 gives the theoretical proofs of main results. The simulations are presented to show the performances of parameter estimates and the M-test for the powers in Section 4, and the conclusions are given in Section 5.

## Main results

In this paper, let *ρ* be a non-monotonic convex function on $\mathbb{R}$, and denote $\psi_{+}$ and $\psi_{-}$ as the right and left derivatives of the function *ρ*, respectively. The derivative function *ψ* is chosen to satisfy $\psi_{-}(u) \leq\psi(u) \leq \psi _{+}(u)$, for all $u \in\mathbb{R}$.

Now, several assumptions are listed as follows:

1. The function $G(u)= E\psi(e_{t}+u)$ exists with $G(0)=E\psi (e_{t})=0$, and has a positive derivative *λ* at $u=0$.
2. $0< E\psi^{2}(e_{1})=\sigma^{2}<\infty$, and $\lim_{u\rightarrow0}E|\psi(e_{1}+u)-\psi(e_{1})|^{2}=0$.
3. There exists a positive constant Δ such that for $h\in (0,\Delta)$, the function $\psi(u+h)-\psi(u)$ is monotonic on *u*.
4. $\sum_{t=2}^{\infty} | \operatorname{cov}(\psi(e_{1}), \psi (e_{t}))|<\infty$.
5. Denote $\mathbf{S}_{n}=\sum_{t=1}^{n}\mathbf {x}_{t}\mathbf{x}_{t}^{T}$, assume that $\mathbf{S}_{n}>0$ for sufficiently large *n*, and
  $$d_{n}= \max_{1\leq t \leq n}\mathbf{x}_{t}^{T} \mathbf {S}_{n}^{-1}\mathbf{x}_{t}=O \bigl(n^{-1}\bigr). $$

### Remark 1

(A1)-(A4) are often applied in the asymptotic theory of M-estimate in regression models (see [20–30]). (A5) is reasonable because it is equivalent to the bound of $\max_{1\leq t \leq n}|\mathbf{x}_{t}\mathbf{x}_{t}^{T}|$, and here is a particular case of the condition $d_{n}=O(n^{-\delta})$ for some $0<\delta\leq1$, which was used in Wang *et al.* [19]. Those functions were mentioned in (2) whose ‘derivative’ function *ψ* correspond to least square (LS) estimate with $\psi(x)=x$, least absolute deviation (LAD) estimate with $\psi(x)=\operatorname{sign}(x)$ and Huber estimate with $\psi(x)=-kI(x<-k)+xI(|x|\leq k)+kI(x>k)$ are satisfied with the above conditions.

### Theorem 1

*In the model* (1), *assume that* $\{e_{t},1\leq t \leq n\}$, *which is a sequence of identically distributed NSD random variables*, *is an uniformly integral family on L*2-*norm*, *and* (A1)-(A5) *hold*. *Then* $2\lambda\sigma^{-2} M_{n}$ *has an asymptotic non*-*central chi*-*squared distribution with* *p*-*degrees of freedom and a non*-*central parameter* $v(n)$, *namely*,

$$2\lambda\sigma^{-2} M_{n} \xrightarrow{\mathfrak{D}} \chi_{p,v(n)}^{2}, $$

*where* $v(n)=\lambda^{2}\sigma^{-2}\|{\boldsymbol {\omega}}(n)\|^{2}$, $\boldsymbol {\omega}(n)=\mathbf{H}_{n}^{\top}\mathbf {S}_{n}^{1/2}\boldsymbol {\omega}_{n}$, $\mathbf{H}_{n}=\mathbf {S}_{n}^{-1/2}\mathbf{H}(\mathbf{H}^{\top}\mathbf {S}_{n}^{-1}\mathbf{H})^{-1/2}$. *In particular*, *when the local alternatives* $\|\mathbf {S}_{n}^{1/2}\boldsymbol {\omega}_{n}\|\rightarrow0$, *which means that the true parameters deviate from the null hypothesis slightly*, *then* $2\lambda\sigma^{-2} M_{n}$ *has an asymptotic central chi*-*squared distribution with* *p* *degrees of freedom*

$$2\lambda\sigma^{-2} M_{n} \xrightarrow{\mathfrak{D}} \chi_{p}^{2}. $$

*For a given significance level* *α*, *we can determine the rejection region as follows*:

7 $$ W=\bigl(0,\chi_{p}^{2}({1-\alpha/2})\bigr) \cup\bigl(\chi_{p}^{2}(\alpha /2),+\infty\bigr), $$

*where* $\chi_{p}^{2}(1-\alpha/2)$, $\chi_{p}^{2}(\alpha/2)$ *are the* $(1-\alpha/2)$-*quantile and* $\alpha/2$-*quantile of central chi*-*squared distribution with* *p* *degrees of freedom*, *respectively*.

### Theorem 2

*Denote*

$$\begin{aligned}& \hat{\sigma}_{n}^{2}=n^{-1}\sum _{t=1}^{n} \psi^{2}\bigl(y_{t}- \mathbf{x}_{t}^{\top}\hat{\boldsymbol {\beta}_{n}}\bigr), \\& \hat{\lambda}_{n}=(2nh)^{-1}\sum _{t=1}^{n} {\psi\bigl(y_{t}- \mathbf{x}_{t}^{\top}\hat{\boldsymbol {\beta }}_{n}+h \bigr)}-{\psi\bigl(y_{t}- \mathbf{x}_{t}^{\top}\hat{ \boldsymbol {\beta}}_{n}-h\bigr)}, \end{aligned}$$

*where* $h=h_{n}>0$, *and* $h_{n}$ *is a sequence chosen to satisfy*

8 $$ h_{n}/d_{n}^{1/2}\rightarrow\infty, \qquad h_{n}\rightarrow0,\qquad \lim_{n\rightarrow\infty}nh_{n}^{2}>0. $$

*Under the conditions of Theorem* 1, *we have*

9 $$\begin{aligned}& \hat{\sigma}_{n}^{2} \xrightarrow{\mathbb{P}} \sigma^{2}, \end{aligned}$$

10 $$\begin{aligned}& \hat{\lambda}_{n}^{2} \xrightarrow{ \mathbb{P}}\lambda. \end{aligned}$$

*Under the assumption* $\|\mathbf{S}_{n}^{1/2}\boldsymbol {\omega }_{n}\|\rightarrow0$, *replacing* *λ*, $\sigma^{2}$ *by their consistent estimates* $\hat{\lambda}_{n}$ *and* $\hat{\sigma }_{n}^{2}$, *then*

$$2\hat{\lambda}_{n} \hat{\sigma}_{n}^{-2} M_{n} \xrightarrow{\mathfrak{D}}\chi_{p}^{2}. $$

## Proof of theorems

It is convenient to consider the rescaled model

11 $$ y_{nt}=\mathbf{x}_{nt}^{\top}\boldsymbol { \beta }(n)+e_{t},\quad t=1,2,\ldots,n, $$

where $\mathbf{x}_{nt}=\mathbf{S}_{n}^{-1/2}\mathbf{x}_{t}$, $\boldsymbol {\beta}(n)=\mathbf{S}_{n}^{1/2}(\boldsymbol {\beta} -\mathbf{b})$, $y_{nt}=y_{t}-\mathbf{x}_{t}^{\top }\mathbf {b}$. It is easily to check that

12 $$ \sum_{t=1}^{n} \bigl\Vert \mathbf{x}_{nt}\mathbf{x}_{nt}^{\top } \bigr\Vert =p. $$

Assume that $q< p$, there exists a $p\times(p-q)$ matrix **K** with the rank $(p-q)$ such that $\mathbf{H}^{\top }\mathbf {K}=\mathbf{0}$ and $\mathbf{K}^{\top}\boldsymbol {\omega }_{n}=\mathbf{0}$, then, for some $\boldsymbol {\gamma}\in \mathbb {R}^{p-q}$, ${H}_{0}$ and ${H}_{2,n}$ can be written as

13 $$ {H}_{0}: \boldsymbol {\beta}-\mathbf{b}=\mathbf {K}\boldsymbol { \gamma}, \qquad {H}_{2,n}:\boldsymbol {\beta}-\mathbf {b}=\mathbf {K}\boldsymbol { \gamma}+\boldsymbol {\omega}_{n}. $$

Denote $\mathbf{H}_{n}=\mathbf {S}_{n}^{-1/2}\mathbf{H}(\mathbf{H}^{\top}\mathbf {S}_{n}^{-1}\mathbf{H})^{-1/2}$, $\mathbf {K}_{n}=\mathbf {S}_{n}^{1/2}\mathbf{K}(\mathbf{K}^{\top}\mathbf {S}_{n}\mathbf{K})^{-1/2}$, then

14 $$ \mathbf{H}_{n}^{\top}\mathbf{H}_{n}= \mathbf {I}_{q}, \qquad\mathbf{K}_{n}^{\top}\mathbf {K}_{n}=\mathbf{I}_{p-q},\qquad \mathbf{H}_{n}^{\top} \mathbf{K}_{n}=\mathbf {0},\qquad \mathbf {H}_{n} \mathbf{H}_{n}^{\top}+\mathbf{K}_{n}\mathbf {K}_{n}^{\top}= \mathbf{I}_{p}. $$

Let $\boldsymbol {\gamma}_{0}(n)=(\mathbf {K}^{\top }\mathbf{S}_{n}\mathbf{K})^{1/2}\boldsymbol {\gamma}$. Under the null hypothesis, model (11) can be rewritten as

$$y_{nt}=\mathbf{x}_{nt}^{\top}\mathbf{K}_{n} \boldsymbol {\gamma }_{0}(n)+e_{t},\quad t=1,2,\ldots,n. $$

Set $\boldsymbol {\omega}(n)=\mathbf{H}_{n}^{\top}\mathbf {S}_{n}^{1/2}\boldsymbol {\omega}_{n}$, $\boldsymbol {\gamma}(n)=\boldsymbol {\gamma}_{0}(n)+\mathbf {K}_{n}^{\top}\mathbf{S}_{n}^{1/2}\boldsymbol {\omega}_{n}$, under the local alternatives (13),

15 $$ \boldsymbol {\beta}(n)=\mathbf {K}_{n}\boldsymbol {\gamma}(n)+ \mathbf{H}_{n}\boldsymbol {\omega}(n). $$

Define $\hat{\boldsymbol {\beta}}(n)=\mathbf{S}_{n}^{1/2} (\hat{\boldsymbol {\beta}}-\mathbf{b})$, and $\hat{\boldsymbol {\gamma }}(n)$ satisfies

$$\min_{\varsigma\in\mathbb{R}^{p-q}}\sum_{t=1}^{n} \rho\bigl(y_{nt}-\mathbf{x}_{nt}^{\top} \mathbf{K}_{n}\boldsymbol {\zeta}\bigr)=\sum_{t=1}^{n} \rho\bigl(y_{nt}-\mathbf{x}_{nt}^{\top} \mathbf{K}_{n}\hat {\boldsymbol {\gamma}}(n)\bigr). $$

Obviously, $\hat{\boldsymbol {\beta}}(n)$, $\hat{\boldsymbol {\gamma }}(n)$ are the M-estimates of $\boldsymbol {\beta}(n)$ and $\boldsymbol {\gamma}(n)$, respectively. Thus

$$\tilde{\boldsymbol {\beta}}=\mathbf{b}+\mathbf {S}_{n}^{-1/2} \mathbf{K}_{n}\hat{\boldsymbol {\gamma}}(n). $$

Next, we will state some lemmas that are needed in order to prove the main results of this paper.

### Lemma 1

Hu [10]

*If* $\{X_{n},n\geq1\}$ *is a NSD random sequence*, *we have the following properties*.

- *For any* $x_{1},x_{2},\ldots,x_{n}$,
  $$P(X_{1} \leq x_{1}, X_{2} \leq x_{2}, \ldots, X_{n} \leq x_{n})\leq\prod _{t=1}^{n} P(X_{t} \leq x_{t}). $$
- *If* $f_{1}, f_{2}, \ldots, f_{n}$ *are non*-*decreasing functions*, *then* $\{f_{n}(X_{n}),n\geq1\}$ *is still a NSD random sequence*.
- *The sequence* $\{-X_{1},-X_{2},\ldots,-X_{n}\}$ *is also NSD*.

### Lemma 2

(Rosenthal inequality) (Shen *et al.* [17])

*Let* $\{X_{n},n\geq1\}$ *be a NSD random sequence with* $E X_{t}=0$ *and* $E|X_{n}|^{p} < \infty$ *for some* $p\geq2$, *then*, *for all* $n\geq1$ *and* $p\geq2$,

$$E \Biggl(\max_{1\leq j \leq n} \Biggl\vert \sum _{t=1}^{j}X_{t} \Biggr\vert ^{p} \Biggr) \leq C \Biggl\{ \sum_{t=1}^{n}E |X_{t}|^{p}+ \Biggl(\sum_{t=1}^{n}E X_{t}^{2} \Biggr)^{p/2} \Biggr\} . $$

### Lemma 3

Anderson *et al.* [31]

*Let* *D* *be an open convex subset of* $\mathbb{R}^{n}$ *and* $\{f_{n}\}$ *are convex functions on* *D*, *for any* $x\in D$,

$$f_{n}(x)\xrightarrow{\mathbb{P}}f(x). $$

*If* *f* *is a real function on* *D*, *then* *f* *is also convex*, *and for all compact subset* $D_{0}\subset D$,

16 $$ \sup_{x\in D_{0}} \bigl\vert f_{n}(x)-f(x) \bigr\vert \xrightarrow{\mathbb{P}} 0. $$

*Moreover*, *if* *f* *is a differentiable function on* *D*, $g(x)$ *and* $g_{n}(x)$ *represent the gradient and sub*-*gradient of* *f*, *respectively*, *then* (16) *implies that for all* $D_{0}$

$$\sup_{x\in D_{0}} \bigl\vert g_{n}(x)-g(x) \bigr\vert \xrightarrow{\mathbb{P}} 0. $$

### Lemma 4

*Assume that* $\{X_{n},n\geq1\}$ *is a sequence of identically distributed NSD random sequence with finite variance*, *and an array of real numbers* $\{a_{nj},1\leq j\leq n\}$ *is satisfied* $\sum_{j=1}^{n}a_{nj}^{2}=O(1)$, $\max_{1\leq j \leq n}|a_{nj}|\rightarrow0$. *Then*, *for any real* $r_{j}$, $j=1,\ldots,n$,

$$\Biggl\vert E \exp\Biggl(i \sum_{j=1}^{n}r_{j} Z_{nj}\Biggr)-\prod_{j=1}^{n}E \exp(ir_{j}Z_{nj}) \Biggr\vert \leq \frac{1}{2}\sum _{j\neq l, j,l=1}^{n} \bigl\vert r_{j}r_{l} \operatorname{Cov}(Z_{nj}, Z_{nl}) \bigr\vert , $$

*where* $Z_{nj}=\sum_{l\in\Upsilon _{j}}a_{nl}X_{l}$, $\{\Upsilon_{j}\}$ *are disjoint subsets of* $\{1,2,\ldots ,n\}$, *i* *refers to imaginary unit*.

### Proof

For a pair of NSD random variables *X*, *Y*, by the property (a) in Lemma 1, we have

17 $$ H(x,y)=P(X\leq x,Y\leq y)-P(X\leq x)P(Y\leq y)\leq0. $$

Denote by $F(x,y)$ the joint distribution functions of $(X,Y)$, and $F_{X}(x)$, $F_{Y}(y)$ the marginal distribution function of *X*, *Y*, one gets

18 $$\begin{aligned} \operatorname{Cov}(X,Y) =& E(XY)-E(X)E(Y)= \iint \bigl[F(x,y)-F_{X}(x)F_{Y}(y) \bigr]\, \mathrm{d}x \,\mathrm{d}y \\ =& \iint H(x,y)\,\mathrm{d}x \,\mathrm{d}y. \end{aligned}$$

Form (18), we obtain

$$\operatorname{Cov}\bigl(f(X),g(Y)\bigr)= \iint f^{\prime} (X)g^{\prime}(Y)H(x,y)\,\mathrm{d}x \, \mathrm{d}y, $$

where $f,g$ are complex valued functions on $\mathbb{R}$ with $f^{\prime}$, $g^{\prime}<\infty$. Combining (17) and (18) yields

19 $$ \bigl\vert \operatorname{Cov}\bigl(f(X),g(Y)\bigr) \bigr\vert \leq \iint \bigl\vert f^{\prime}(X) \bigr\vert \bigl\vert g^{\prime}(Y) \bigr\vert \bigl\vert H(x,y) \bigr\vert \,\mathrm{d}x \,\mathrm{d}y \leq \bigl\Vert f^{\prime} \bigr\Vert _{\infty} \bigl\Vert g^{\prime} \bigr\Vert _{\infty} \bigl\vert \operatorname{Cov}(X,Y) \bigr\vert . $$

Taking $f(X)=\exp(irX)$, $g(Y)=\exp(iuY)$, it is easily seen that

$$\bigl\Vert f^{\prime}(X) \bigr\Vert _{\infty} \leq1 < \infty, \qquad \bigl\Vert g^{\prime}(Y) \bigr\Vert _{\infty} \leq1 < \infty, $$

thus for any real numbers *r*, *u*

20 $$ \bigl\vert E\exp(irX+iuY)-E\exp(irX)E\exp(iuY) \bigr\vert \leq \bigl\vert ru\operatorname{Cov}(X,Y) \bigr\vert . $$

Next, we proceed the proof by induction on *n*. Lemma 4 for $n=1$ is trivial and for $n=2$ is true by (20). Assume that the result is true for all $n\leq M$ ($n\geq3$). For $n=M+1$, there exist some $\epsilon ^{2}=1$, $\delta^{2}=1$, $k\in\{1,\ldots,M\}$ such that

$$\textstyle\begin{cases} \epsilon r_{j} \geq0, & 1 \leq j \leq k,\\ \delta r_{j} \geq0,& 1+k \leq j \leq M+1. \end{cases} $$

Denote $X=\sum_{j=1}^{k}\epsilon r_{j}Z_{nj}$, $Y=\sum_{j=k+1}^{M+1}\delta r_{j}Z_{nj}$, then

$$\sum_{j=1}^{n}r_{j}Z_{nj}= \epsilon X+\delta Y. $$

Note that $X,Y$ are non-decreasing functions, we have by the induction hypothesis that

$$\begin{aligned}& \Biggl\vert E\exp\Biggl(i\sum_{j=1}^{n}r_{j}Z_{nj} \Biggr)- \prod_{j=1}^{n}E \exp(ir_{j}Z_{nj}) \Biggr\vert \\ & \quad\leq \bigl\vert E(T_{1}T_{2})-E(T_{1})E(T_{2}) \bigr\vert + \Biggl\vert E(T_{1})E(T_{2})-E(T_{1}) \prod_{j=1+k}^{M+1}E(R) \Biggr\vert \\& \qquad {}+ \Biggl\vert E(T_{1})\prod_{j=1+k}^{M+1}E(R)- \prod_{j=1}^{k}E(R) \Biggr\vert \\& \quad\leq \vert \epsilon\delta \vert \bigl\vert \operatorname{Cov}(X,Y)\bigr|+\Bigg|E(T_{2})- \prod_{j=1+k}^{M+1}E(R) \Biggr\vert + \Biggl\vert E(T_{1})-\prod_{j=1+k}^{M+1}E(R) \Biggr\vert \\& \quad\leq \Biggl\vert \operatorname{Cov}\Biggl(\sum _{j=1}^{k}\epsilon r_{j}Z_{nj}, \sum_{j=k+1}^{M+1}\delta r_{l}Z_{nl} \Biggr) \Biggr\vert + \frac{1}{2}\sum_{j\neq l, j,l=k+1}^{M+1} \bigl\vert r_{j} r_{l} \operatorname{Cov}(Z_{nj}, Z_{nl}) \bigr\vert \\& \qquad{}+\frac{1}{2}\sum _{j\neq l, j,l=1}^{M+1} \bigl\vert r_{j} r_{l} \operatorname{Cov}(Z_{nj}, Z_{nl}) \bigr\vert \\& \quad\leq \frac{1}{2}\sum_{j\neq l, j,l=1}^{n} \bigl\vert r_{j} r_{l} \operatorname{Cov}(Z_{nj}, Z_{nl}) \bigr\vert , \end{aligned}$$

where $T_{1}=\exp(i\epsilon X)$, $T_{2}=\exp(i\delta Y)$, $R=\exp (ir_{j} Z_{nj})$. Thus, this completes the proof of Lemma 4. □

### Lemma 5

Billingsley [32]

*If* $X_{nj}\xrightarrow{L} X_{j}$, $X_{j} \xrightarrow{L} X$ *for each* *j*, *and uniform measure* *ϱ* *is satisfied that for all* $\varepsilon>0$,

$$\lim_{j\rightarrow\infty}\lim_{n\rightarrow \infty}\sup\bigl\{ \varrho(X_{nj},Y_{n})\geq\varepsilon\bigr\} =0, $$

*then*

$$Y_{n}\xrightarrow{L} X. $$

### Lemma 6

*Suppose that* $\{X_{n},n\geq1\}$ *and* $\{a_{nj},1\leq j\leq n\}$ *satisfy the assumptions of Lemma* 4. *Further assume that* $\{X_{n},n\geq1\}$ *is an uniformly integral family on* $L_{2}$-*norm*, *then*

$$\sigma_{n}^{-1}\sum_{j=1}^{n}a_{nj}X_{j} \xrightarrow{\mathfrak{D}} \mathrm{N}(0,1), $$

*where* $\sigma_{n}^{2}=\operatorname{var} (\sum_{j=1}^{n}a_{nj}X_{j} )$.

### Proof

Without loss of generality, we suppose that $a_{nj}=0$ for all $j>n$. By (18), we have $\operatorname{Cov}(X,Y)\leq 0$ because of the negativity of $H(x,y)$. Then, for $1\leq m\leq n-1$,

$$\begin{aligned} \sum_{l,j=1,|l-j|\geq m}^{n} \bigl\vert a_{nl}a_{aj}\operatorname{Cov}(X_{l},X_{j}) \bigr\vert \leq& \sum_{j=1}^{n-u}\sum _{l=j+u}^{n}\bigl(a_{nj}^{2}+a_{nl}^{2} \bigr) \bigl\vert \operatorname{Cov}(X_{l},X_{j}) \bigr\vert \\ \leq & \sum_{j=1}^{n-m}a_{nj}^{2} \sum_{l=j+ m}^{n} \bigl\vert \operatorname{Cov}(X_{l},X_{j}) \bigr\vert +\sum _{l=j+ m}^{n}a_{nj}^{2}\sum _{j=1}^{l-m} \bigl\vert \operatorname{Cov}(X_{l},X_{j}) \bigr\vert \\ \leq & \sum_{j=1}^{n}a_{nj}^{2} \sum_{|l-j|\geq m}^{n} \bigl\vert \operatorname{Cov}(X_{l},X_{j}) \bigr\vert \\ \leq & \sup_{j} \Biggl\vert \sum _{l=1,|l-j|\geq m}^{n} \operatorname{Cov}(X_{l}, X_{j}) \Biggr\vert \Biggl(\sum_{l=1}^{n}a_{nl}^{2} \Biggr). \end{aligned}$$

Taking $\psi(x)=x$ in assumption (A4), we get, for all $l\geq1$ and sufficiently large *j*,

$$\sum_{j:|l-j|\geq m}^{\infty} \bigl\vert \operatorname{Cov}(X_{l},X_{j}) \bigr\vert \rightarrow0. $$

Therefore, for a fixed small *ε*, there exists a positive integer $m=m_{\varepsilon}$ such that

21 $$ \sum_{l,j=1, |l-j|\geq m} \bigl\vert a_{nl}a_{nj}\operatorname{Cov}(X_{l},X_{j}) \bigr\vert \leq \varepsilon. $$

Denote $N_{0}=[1/\varepsilon]$, where $[x]$ denotes the integer part of *x*, and $Y_{nj}=\sum_{k=mj+1}^{m(j+1)}a_{nk}X_{k}$, $j=0,1,2,\ldots,n$,

$$\Upsilon_{j}=\Biggl\{ j:2 N_{0}l \leq j \leq2N_{0}l+N_{0}, \bigl\vert \operatorname{Cov}(Y_{nj},Y_{nj+1}) \bigr\vert \leq \frac{2}{N_{0}}\sum_{j=2N_{0}l}^{2N_{0}l+N_{0}} \operatorname{Var}(Y_{nl})\Biggr\} . $$

We define $s_{0}=0$, $s_{j+1}=\min\{s:s>s_{j}, s\in\Upsilon_{j}\}$, and put

$$\begin{aligned}& Z_{nj}=\sum_{l=s_{j}+1}^{s_{j+1}} Y_{nl}, \quad j=0,1,2,\ldots,n, \\& \Lambda_{j}= \bigl\{ m(s_{j}+1)+1,\ldots, m(s_{j+1}+1) \bigr\} . \end{aligned}$$

Note that

$$Z_{nj}=\sum_{l\in\Lambda_{j}}a_{nl}X_{l}, \quad j=0,1,2,\ldots,n, $$

it is easy to see that $\# \Lambda_{j}\leq3 N_{0} m$, where # stands for the cardinality of a set. Next, we will proof that $\{Z_{nj},1\leq j \leq n\}$ satisfies the Lindeberg condition.

Let $B_{n}^{2}=\sum_{j=1}^{n}E Z_{nj}^{2}$, by Lemma 2, it yields

$$\begin{aligned} B_{n}^{2} =& \sum_{j=1}^{n}E \biggl(\sum_{l\in\Lambda _{j}}a_{nl}X_{l} \biggr)^{2} \\ \leq & \sum_{j=1}^{n}a_{nl}^{2}E \biggl(\sum_{l\in \Lambda _{j}}X_{l} \biggr)^{2} \\ \leq & \sum_{j=1}^{n}\sum _{l\in\Lambda _{j}}a_{nl}^{2}E(X_{l})^{2} \\ \leq & \sum_{j=1}^{n}E(a_{nl}X_{j})^{2}= \sigma_{n}^{2}< \infty. \end{aligned}$$

Clearly, $\{Z_{nj}^{2}\}$ is uniform integrable since $\{ X_{j}^{2}, j\geq1 \}$ is uniform integrable. Hence, for any positive *ε*, $\{Z_{nj},1\leq j \leq n\}$ is verified to satisfy the Lindeberg condition by

$$\begin{aligned} \frac{1}{B_{n}^{2}}\sum_{j=1}^{n}E Z_{nj}^{2}I \bigl\{ \vert Z_{nj} \vert \geq & \varepsilon B_{n} \bigr\} \leq\sum_{j=1}^{n} \biggl(\sum_{l\in \Lambda_{j}}a_{nl}^{2} \biggr)\max_{l\in\Lambda_{j}} E X_{l}^{2} I \biggl\{ \sum_{l\in\Lambda_{j}} \vert X_{l} \vert \geq \varepsilon/ \max_{l\in\Lambda_{j}}|a_{nl}| \biggr\} \\ \leq & \Biggl(\sum_{j=1}^{n}a_{nl}^{2} \Biggr)\max_{1\leq j \leq n} E X_{l}^{2} I \biggl\{ \sum_{l\in \Lambda_{j}}X_{l}^{2}\geq \varepsilon^{2} / \Bigl(\max_{l\in\Lambda_{l}} \vert a_{nl} \vert \Bigr)^{2} \biggr\} \\ \leq & \Biggl(\sum_{j=1}^{n}a_{nl}^{2} \Biggr)\max_{1\leq j \leq n} E \sum_{l\in\Lambda_{j}}X_{l}^{2} I \biggl\{ \sum_{l\in\Lambda_{j}}X_{l}^{2} \geq\varepsilon^{2} / \Bigl(\max_{l\in\Lambda_{j}} \vert a_{nl} \vert \Bigr)^{2} \biggr\} . \end{aligned}$$

Now taking an independence random sequence $\{Z_{nj}^{*},j=1,2,\ldots ,n\}$ such that have same marginal distribution with $Z_{nj}$ for each *j*. Let $F(Z_{n1},Z_{n2},\ldots ,Z_{nn})$ and $G(Z_{n1}^{*},Z_{n2}^{*},\ldots,Z_{nn}^{*})$ be the eigenfunctions of $\sum_{j=1}^{n}Z_{nj}$ and $\sum_{j=1}^{n}Z_{nj}^{*}$, respectively. Choosing $r=\max\{r_{l},r_{j}\}$, we have by Lemma 4 and (21)

$$\begin{aligned} \vert F-G \vert = & \Biggl\vert E\exp \Biggl(i \sum _{j=1}^{n} r_{j} Z_{nj} \Biggr)- E\exp \Biggl(i \sum_{j=1}^{n} r_{j} Z_{nj}^{*} \Biggr) \Biggr\vert \\ =& \Biggl\vert E\exp \Biggl(i \sum_{j=1}^{n} r_{j} Z_{nj} \Biggr)- \prod_{j=1}^{n}E \exp(ir_{j}Z_{nj}) \Biggr\vert \\ \leq & \frac{1}{2}\sum_{j\neq l, j,l=1}^{n} \bigl\vert r_{j} r_{l} \operatorname{Cov}(Z_{nj},Z_{nl}) \bigr\vert \\ \leq & r^{2} \Biggl(\sum_{1\leq l < j\leq n, |l-j|=1}^{n} \bigl\vert \operatorname{Cov}(Z_{nj},Z_{nl}) \bigr\vert + \sum_{1\leq l < j\leq n, |l-j|>1}^{n} \bigl\vert \operatorname{Cov}(Z_{nj},Z_{nl}) \bigr\vert \Biggr) \\ \leq & r^{2} \Biggl(\sum_{j=1}^{n} \bigl\vert \operatorname{Cov}(Y_{ns_{l}},Y_{ns_{l+1}}) \bigr\vert +\sum_{1\leq l < j\leq n, |l-j|>m}^{n} \vert a_{nl}a_{nj} \vert \bigl\vert \operatorname{Cov}(X_{nj},X_{nl}) \bigr\vert \Biggr) \\ \leq & r^{2} \Biggl(\frac{C}{N_{0}}\sum _{j=1}^{n}\operatorname{Var}(Y_{nj})+ \varepsilon \Biggr) \leq\varepsilon\bigl(r^{2}+C \sigma_{n}^{2} \bigr). \end{aligned}$$

By Levy’s theorem, ${Z_{nj}^{*}}$ obtains the asymptotic normality, applying Lemma 5, then the identically distribution property of $\{ X_{j}\}$ implies that

$$B_{n}^{-1}\sum_{j=1}^{n}Z_{nj}= \sigma_{n}^{-1}\sum_{j=1}^{n}a_{nj}X_{j} \xrightarrow{\mathfrak{D}} \mathrm{N}(0,1), $$

which completes the proof of Lemma 6. □

### Lemma 7

*In the model* (1), *assume that* $\{e_{t},1\leq t \leq n\}$ *is a sequence of NSD identically distributed random variables*, (A1)-(A4) *are satisfied*, *for any positive constant* *δ* *and sufficiently large* *n*, *then*

$$\begin{aligned}& \sup_{| \boldsymbol {\tau} | \leq\delta} \Biggl\vert \sum_{t=1}^{n} \bigl\{ \rho\bigl(e_{t}-\mathbf{x}_{nt}^{\top}\boldsymbol { \tau}\bigr) -\rho(e_{t})+\psi(e_{t})\mathbf{x}_{nt}^{\top} \boldsymbol {\tau}^{\top} \bigr\} -\frac{1}{2}\lambda\boldsymbol { \tau}^{\top} \boldsymbol {\tau} \Biggr\vert \xrightarrow{\mathbb{P}}0, \\& \sup_{| \boldsymbol {\tau} | \leq\delta} \Biggl\vert \sum_{t=1}^{n} \bigl\{ \psi\bigl(e_{t}-\mathbf{x}_{nt}^{\top} \boldsymbol { \tau}\bigr)-\psi(e_{t}) \bigr\} \mathbf{x}_{nt}^{\top}+ \lambda\boldsymbol {\tau} \Biggr\vert \xrightarrow{\mathbb{P}}0, \end{aligned}$$

*where* ***τ*** *is a* *p*-*vector*.

### Proof

Denote

$$\begin{aligned} f_{n}(\boldsymbol {\tau}) = & \sum_{t=1}^{n} \bigl\{ \rho\bigl(e_{t}-\mathbf{x}_{nt}^{\top}\boldsymbol { \tau}\bigr) -\rho(e_{t})+\psi(e_{t})\mathbf{x}_{nt}^{\top} \boldsymbol {\tau} \bigr\} \\ = & \sum_{t=1}^{n} \int_{0}^{-\mathbf{x}_{nt}^{\top }\boldsymbol {\tau}} \bigl\{ \psi(e_{t}+u)- \psi(e_{t}) \bigr\} \,du. \end{aligned}$$

For fixed ***τ***, it follows from (A5) that

22 $$ \max_{1 \leq t \leq n} \bigl\vert \mathbf{x}_{nt}^{\top} \boldsymbol {\tau } \bigr\vert \rightarrow O\bigl(n^{-1/2}\bigr). $$

From (A1) and (22), there exist a sequence of positive numbers $\varepsilon_{n}\rightarrow0$ and $\theta_{nt}\in(-1,1)$ such that, for sufficiently large *n*,

$$\begin{aligned} Ef_{n}(\boldsymbol {\tau}) = & \sum _{t=1}^{n} \int _{0}^{-\mathbf{x}_{nt}^{\top} \boldsymbol {\tau}}E \bigl(\psi(e_{t}+u)- \psi(e_{t})\bigr)\,du \\ = & \sum_{t=1}^{n} \int_{0}^{-\mathbf{x}_{nt}^{\top}\boldsymbol {\tau}}\bigl\{ \lambda u+o\bigl( \vert u \vert \bigr)\bigr\} \,du \\ = & \frac{1}{2}\lambda\sum_{t=1}^{n} \bigl(\mathbf {x}_{nt}^{\top }\boldsymbol {\tau}\bigr)^{2} (1+ \varepsilon_{n}\theta_{nt})\rightarrow \frac{1}{2} \lambda\boldsymbol {\tau}^{\top}\boldsymbol {\tau}. \end{aligned}$$

In view of the monotonicity of $\psi(e_{t}+u)-\psi(e_{t})$, the summands of $f_{n}(\boldsymbol {\tau})$ is also monotonous with respect to $e_{t}$ from the property (b) in Lemma 1. We divide the summands of $f_{n}(\boldsymbol {\tau})$ into positive and negative two parts, by the property (c) in Lemma 1, they are still NSD. Therefore, applying Schwarz’s inequality and (22), we obtain

$$\begin{aligned} \operatorname{var}\bigl[f_{n}(\boldsymbol {\tau})\bigr] =& E \Bigg\{ \sum _{t=1}^{n} \biggl[ \int_{0}^{-\mathbf{x}_{nt}^{\top}\boldsymbol {\tau}} \bigl(\psi(e_{t}+u)- \psi(e_{t})\bigr)\,du \biggr]^{+} \\ &{}-\sum_{t=1}^{n} \biggl[ \int_{0}^{-\mathbf{x}_{nt}^{\top}\boldsymbol {\tau}} \bigl(\psi(e_{t})+u\bigr)- \psi(e_{t}) \biggr]^{-}\,du \Bigg\} ^{2} \\ \leq& E \Biggl\{ \sum_{t=1}^{n} \biggl[ \int_{0}^{-\mathbf{x}_{nt}^{\top}\boldsymbol {\tau}} \bigl(\psi(e_{t}+u)- \psi(e_{t})\bigr)\,du \biggr]^{+} \Biggr\} ^{2} \\ &{}+E \Biggl\{ \sum_{t=1}^{n} \biggl[ \int_{0}^{-\mathbf{x}_{nt}^{T}\boldsymbol {\tau}} \bigl(\psi(e_{t}+u)- \psi(e_{t})\bigr)\,du \biggr]^{-} \Biggr\} ^{2} \\ \leq& \sum_{t=1}^{n} E \biggl[ \int_{0}^{-\mathbf {x}_{nt}^{\top}\boldsymbol {\tau}} \bigl(\psi(e_{t}+u)- \psi(e_{t})\bigr)\,du \biggr]^{2} \\ \leq& \sum_{t=1}^{n} | \mathbf{x}_{nt}^{\top}| \biggl\vert \int _{0}^{-\mathbf{x}_{nt}^{\top}\boldsymbol {\tau}} E\bigl[\psi(e_{t}+u)- \psi(e_{t})\bigr]^{2}\,du \biggr\vert \\ =&o(1)\sum_{t=1}^{n} \bigl( \mathbf{x}_{nt}^{\top }\bigr)^{2}\rightarrow0. \end{aligned}$$

Hence for sufficiently large *n*, we have

23 $$ f_{n}(\boldsymbol {\tau})\xrightarrow{\mathbb{P}} \frac{1}{2}\lambda\boldsymbol {\tau}^{\top}\boldsymbol {\tau}. $$

Lemma 7 is proved by (23) and Lemma 3. □

### Lemma 8

*Under conditions of Lemma* 7 *and the local alternatives* (5)-(6), *then we see that*, *for any positive constant* *δ* *and sufficiently large* *n*,

24 $$\begin{aligned}& \sup_{|\boldsymbol {\xi}_{1} | \leq\delta} \Biggl\vert \sum _{t=1}^{n} \bigl\{ \rho\bigl(y_{nt}- \mathbf{x}_{nt}^{\top}\boldsymbol {\eta}\bigr)- \rho(e_{t})+ \psi(e_{t})\mathbf{x}_{nt}^{\top} \boldsymbol { \xi}_{1} \bigr\} -\frac{1}{2}\lambda \Vert \boldsymbol { \xi}_{1} \Vert ^{2} \Biggr\vert \xrightarrow{ \mathbb{P}}0, \end{aligned}$$

25 $$\begin{aligned}& \sup_{|\boldsymbol {\xi}_{1} | \leq\delta} \Biggl\vert \sum _{t=1}^{n} \bigl[\psi\bigl(y_{nt}- \mathbf{x}_{nt}^{\top}\boldsymbol {\eta}\bigr)-\psi(e_{t}) \bigr]\mathbf{x}_{nt}^{\top}+ \lambda\boldsymbol {\xi}_{1} \Biggr\vert \xrightarrow{\mathbb{P}}0, \end{aligned}$$

26 $$\begin{aligned}& \sup_{|\boldsymbol {\xi}_{2} | \leq\delta} \Bigg\vert \sum_{t=1}^{n} \bigl\{ \rho\bigl(y_{nt}-\mathbf{x}_{nt}^{\top}\mathbf {K}_{n}\boldsymbol {\zeta}\bigr) -\rho(e_{t}) \bigr\} +\sum _{t=1}^{n}\psi(e_{t})\mathbf {x}_{nt}^{\top} \bigl(\mathbf{K}_{n}\boldsymbol {\zeta}- \boldsymbol {\beta}(n)\bigr) \\& \quad{}-\frac{1}{2}\lambda \Vert \boldsymbol { \xi}_{2} \Vert ^{2} + \bigl\Vert \boldsymbol {\omega}(n) \bigr\Vert ^{2} \Bigg\vert \xrightarrow{\mathbb{P}}0, \end{aligned}$$

27 $$\begin{aligned}& \sup_{|\boldsymbol {\xi}_{2} | \leq\delta} \Biggl\vert \sum _{t=1}^{n} \bigl[\psi\bigl(y_{nt}- \mathbf{x}_{nt}^{\top}\mathbf {K}_{n}\boldsymbol {\zeta} \bigr)-\psi(e_{t}) \bigr]\mathbf{x}_{nt}+\lambda\bigl( \mathbf{K}_{n}\boldsymbol {\zeta }-\boldsymbol {\beta}(n)\bigr) \Biggr\vert \xrightarrow{\mathbb{P}}0, \end{aligned}$$

*where* $\boldsymbol {\xi}_{1}=\boldsymbol {\eta}-\boldsymbol {\beta}(n)$, $\boldsymbol {\xi}_{2}=\boldsymbol {\zeta}-\boldsymbol {\tau}(n)$.

### Proof

Take the proofs of (24) and (25) as examples, the rest, equations (26) and (27), are the same. Note that (24) can be written as

$$\begin{aligned}& \sup_{|\boldsymbol {\xi}_{2} | \leq\delta} \Bigg\vert \sum_{t=1}^{n} \bigl\{ \rho\bigl(e_{t}-\mathbf{x}_{nt}^{\top}\bigl( \mathbf {K}_{n}\boldsymbol {\zeta}-\mathbf{H}_{n} \boldsymbol {\omega}(n) \bigr)\bigr) -\rho(e_{t}) \bigr\} +\sum_{t=1}^{n} \psi(e_{t})\mathbf {x}_{nt}^{\top} \bigl( \mathbf{K}_{n}\boldsymbol {\zeta}-\mathbf{H}_{n} \boldsymbol {\omega}(n) \bigr) \\& \quad{}-\frac{1}{2}\lambda\|\boldsymbol {\xi}_{2} \|^{2} + \bigl\Vert \boldsymbol {\omega}(n) \bigr\Vert ^{2} \Bigg\vert \xrightarrow{\mathbb{P}}0. \end{aligned}$$

On the other hand, $\|\mathbf{S}_{n}^{1/2}\boldsymbol {\omega }_{n}\| =O(1)$ and $\|\boldsymbol {\gamma}_{2}\|\leq\delta$, hence there exists a constant $\delta_{1}$ such that

28 $$ \bigl\vert \mathbf{K}_{n}\boldsymbol {\zeta}- \mathbf{H}_{n} \boldsymbol {\omega}(n) \bigr\vert \leq\delta_{1}. $$

Thus (24) and (25) follow naturally by (28) and Lemma 7. □

### Lemma 9

*Under the conditions of Theorem* 1, *as* $n\rightarrow \infty$, *we have*

29 $$\begin{aligned}& \hat{\boldsymbol {\beta}}(n)-\boldsymbol {\beta}(n)=\lambda^{-1} \sum_{t=1}^{n}\mathbf{x}_{nt} \psi(e_{t})+o_{P}(1), \end{aligned}$$

30 $$\begin{aligned}& \hat{\boldsymbol {\gamma}}(n)-\boldsymbol {\gamma}(n)=\lambda^{-1} \sum_{t=1}^{n}\mathbf{K}_{n}^{\top} \mathbf {x}_{nt}\psi (e_{t})+o_{P}(1). \end{aligned}$$

### Proof

The estimate of (2) can be defined essentially as the solution of the following equation:

31 $$ \Biggl\Vert \mathbf{S}_{n}^{-1/2}\sum _{t=1}^{n} \psi\bigl(y_{t}- \mathbf{x}_{t}^{\top} \hat{\boldsymbol {\beta}}\bigr) \mathbf{x}_{t} \Biggr\Vert =o_{P}(1). $$

Denote $\hat{\boldsymbol {\beta}}(n)=S_{n}^{1/2} \hat{\boldsymbol {\beta}}_{n}$, (31) can be rewritten to give

32 $$ \Biggl\Vert \sum_{t=1}^{n} \psi\bigl(e_{t}-\mathbf{x}_{nt}^{\top} \hat{\boldsymbol {\beta}}(n)\bigr) \mathbf{x}_{nt} \Biggr\Vert =o_{P}(1). $$

By a routine argument, we shall prove that

33 $$ \bigl\vert \hat{\boldsymbol {\beta}}(n) \bigr\vert =O_{p}(1). $$

Let *U* be a denumerable dense subset in the unit sphere of $\mathbb {R}^{p}$ such that

$$U=\bigl\{ \boldsymbol {\beta}\in\mathbb{R}^{p} : \Vert \boldsymbol {\beta} \Vert =1\bigr\} . $$

Write

$$D(\boldsymbol {\tau}, L)=\sum_{t=1}^{n}\psi \bigl(e_{t}-L\mathbf{x}_{nt}^{T} \boldsymbol {\tau} \bigr) \mathbf{x}_{nt}^{T}\boldsymbol {\tau}, $$

where $L\geq0$, $\boldsymbol {\tau} \in\mathbb{R}^{p}$.

Obviously, for a given ***τ***, $D(\boldsymbol {\cdot}, L)$ is non-decreasing on *L* since *ψ* is non-decreasing. For any $\varepsilon>0$, let

$$L_{0}=\sqrt{p}\sigma/ (\lambda\sqrt{\varepsilon}). $$

Thus by (32), there exists a number $n_{1}$, as $n\geq n_{1}$,

$$\Pr \biggl\{ \biggl\vert D\biggl(\frac{\hat{\boldsymbol {\beta}}(n)}{ \|\hat{\boldsymbol {\beta}}(n)\|}, \bigl\| \hat{\boldsymbol {\beta}}(n)\bigr\| \biggr) \biggr\vert \geq L_{0} \lambda \biggr\} < \varepsilon. $$

Note that $D(\boldsymbol {\tau},\boldsymbol {\cdot})$ is a non-decreasing function on ***τ*** for given *L*, then

$$\Pr \bigl\{ \bigl\Vert \hat{\boldsymbol {\beta}}(n) \bigr\Vert \geq L_{0} \bigr\} < \Pr \Bigl\{ \sup_{\boldsymbol {\tau} \in U}D(\tau, L_{0})\leq-L_{0} \lambda \Bigr\} +\varepsilon. $$

Based on Lemma 8 and $\max_{1\leq t \leq n} |\mathbf{x}_{nt}^{\top}\boldsymbol {\tau}|=O(n^{-1/2})$, one can see that

34 $$ \sup_{\boldsymbol {\tau}\in U} \Biggl\vert \sum _{t=1}^{n}\bigl[\psi\bigl(e_{t}-L_{0} \mathbf {}x_{nt}^{\top} \boldsymbol {\tau}\bigr) -\psi(e_{t}) \bigr]\mathbf{x}_{nt}^{\top}\boldsymbol {\tau}+ L_{0}\lambda \Biggr\vert \rightarrow 0. $$

On the other hand, by Schwarz’s inequality, we have

35 $$ \sup_{\boldsymbol {\tau}\in U} \Biggl\vert \sum _{t=1}^{n}\psi(e_{t}) \mathbf{x}_{nt}^{\top} \boldsymbol {\tau} \Biggr\vert \leq \Biggl\Vert \sum_{t=1}^{n} \psi(e_{t})\mathbf{x}_{nt} \Biggr\Vert . $$

Combining (34) and (35), there exists $n_{2}$ $(n_{1} \leq n_{2}\leq n)$ such that

36 $$ \Pr \Biggl\{ \sup_{\boldsymbol {\tau}\in U} D(\boldsymbol {\tau}, L_{0})< -L_{0}\lambda+ \Biggl\Vert \sum _{t=1}^{n}\psi(e_{t}) \mathbf{x}_{nt} \Biggr\Vert \Biggr\} >1-\varepsilon. $$

Applying Chebyshev’s inequality, the $C_{r}$ inequality and Lemma 8, we have

37 $$\begin{aligned} \Pr \Biggl\{ \Biggl\Vert \sum_{t=1}^{n} \psi(e_{t})\mathbf {x}_{nt} \Biggr\Vert \geq L_{0}\lambda \Biggr\} \leq& \biggl(\frac{1}{L_{0} \lambda} \biggr)^{2} E \Biggl\Vert \sum_{t=1}^{n} \psi(e_{t})\mathbf{x}_{nt} \Biggr\Vert ^{2} \\ \leq& \biggl(\frac{1}{L_{0} \lambda}\biggr)^{2} E \sum _{t=1}^{n} \bigl\Vert \psi(e_{t}) \mathbf{x}_{nt} \bigr\Vert ^{2} \\ \leq& \biggl(\frac{1}{L_{0} \lambda}\biggr)^{2} E \sum _{t=1}^{n} \sigma^{2} \Vert \mathbf{x}_{nt} \Vert ^{2}\leq\varepsilon. \end{aligned}$$

From (36) and (37), it follows that

$$\Pr \Bigl\{ \sup_{\boldsymbol {\tau}\in U} D(\boldsymbol {\tau}, L_{0})< -L_{0} \lambda \Bigr\} >1-2\varepsilon. $$

Likewise, when $n\geq n_{2}$, we obtain

38 $$ \Pr\bigl\{ \bigl\Vert \hat{\boldsymbol {\beta}}(n) \bigr\Vert \geq L_{0}\bigr\} < \varepsilon+1-(1-2\varepsilon)=3\varepsilon. $$

Thus the result (33) follows from (38) and the arbitrariness of *ε*.

By Lemma 8 and $\max_{1\leq t \leq n}|\mathbf{x}_{nt}^{\top}\boldsymbol {\tau}|=O(n^{-1/2})$, it follows that

$$I\bigl( \bigl\Vert \hat{\boldsymbol {\beta}}(n) \bigr\Vert \geq L_{0} \bigr) \Biggl\vert \sum_{t=1}^{n}\bigl[\psi \bigl(e_{t}-\mathbf{x}_{nt}^{\top} \hat{\boldsymbol { \beta}}(n)\bigr)-\psi(e_{t})\bigr]\mathbf{x}_{nt} +\lambda \hat{\boldsymbol {\beta}}(n) \Biggr\vert \xrightarrow{\mathbb{P}} 0, $$

which implies that

$$\hat{\boldsymbol {\beta}}(n)=\lambda^{-1} \sum_{t=1}^{n} \mathbf{x}_{nt}\psi(e_{t})+o_{p}(1). $$

Consequently, (29) is proved.

As defined in (15), $\hat{\boldsymbol {\beta}}(n)$ can similarly be written as $\hat{\boldsymbol {\beta}}(n)=\mathbf{K}_{n}\hat {\boldsymbol {\gamma}}(n)+\mathbf{H}_{n}\boldsymbol {\omega}(n)$, replacing $\boldsymbol {\beta}(n)$, $\hat{\boldsymbol {\beta}}(n)$ by $\boldsymbol {\gamma}(n)$ and $\hat{\boldsymbol {\gamma}}(n)$, respectively, (30) is proved by $\mathbf{K}_{n}^{\top}\mathbf {K}_{n}= \mathbf{I}_{p-q}$. □

### Proof of Theorem 1

According to (33) and Lemma 8, one gets

39 $$ \begin{aligned} & \sum_{t=1}^{n} \bigl[\rho\bigl(y_{nt}-\mathbf{x}_{nt}^{T}\hat{ \boldsymbol {\beta}}(n)\bigr) -\rho(e_{t}) \bigr]+\sum _{t=1}^{n} \psi(e_{t})\mathbf{x}_{nt} \bigl(\hat{\boldsymbol {\beta}}- \boldsymbol {\beta}(n)\bigr)-\frac{\lambda}{2} \bigl\Vert \hat{\boldsymbol {\beta}}-\boldsymbol {\beta}(n) \bigr\Vert ^{2}\xrightarrow{ \mathbb{P}}0, \\ & \sum_{t=1}^{n} \bigl[\rho \bigl(y_{nt}-\mathbf{x}_{nt}^{\top}\hat{\boldsymbol {\beta }}(n)\bigr)-\rho (e_{t}) \bigr] +\frac{1}{2\lambda} \Biggl\Vert \sum _{t=1}^{n} \psi(e_{t}) \mathbf{x}_{nt} \Biggr\Vert ^{2}\xrightarrow{\mathbb{P}}0. \end{aligned} $$

Similarly,

40 $$\begin{aligned}& \sum_{t=1}^{n} \bigl[\rho \bigl(y_{nt}-\mathbf{x}_{nt}^{\top} \mathbf{K}_{n}\hat {\boldsymbol {\gamma}}(n)\bigr)-\rho(e_{t}) \bigr] +\frac{1}{2\lambda} \Biggl\Vert \sum_{t=1}^{n} \psi(e_{t})\mathbf{K}_{n}^{\top}\mathbf{x}_{nt} \Biggr\Vert ^{2} \\& \quad {}-\sum_{t=1}^{n} \psi(e_{t})\mathbf{x}_{nt}^{\top }\mathbf {H}_{n}\boldsymbol {\omega}(n) - \frac{\lambda}{2} \bigl\Vert \boldsymbol { \omega}(n) \bigr\Vert ^{2}\xrightarrow{\mathbb{P}}0. \end{aligned}$$

From (14), (39) and (40), one can see that

41 $$\begin{aligned} 2\lambda\sigma_{n}^{2} M_{n} =& \Biggl\Vert \sum _{t=1}^{n}\mathbf {H}_{n}^{\top} \mathbf{x}_{nt} \psi(e_{t}) \Biggr\Vert ^{2} + \lambda^{2} \bigl\| \boldsymbol {\omega}(n)\bigr\| ^{2} +2 \lambda\boldsymbol { \omega}^{\top }(n)\sum_{t=1}^{n} \mathbf{H}_{n}^{\top} \mathbf{x}_{nt} \psi (e_{t})+o_{P}(1) \\ = & \Biggl\Vert \sum_{t=1}^{n} \mathbf{H}_{n}^{\top} \mathbf {x}_{nt} \psi(e_{t})+ \lambda\boldsymbol {\omega}(n) \Biggr\Vert ^{2}+o_{P}(1). \end{aligned}$$

Since $E\psi(e_{t})=0$, $E\psi^{2}(e_{t})=\sigma^{2} <\infty$, $\max_{1\leq t \leq n}\|\mathbf{x}_{nt}\boldsymbol {\tau}\|=O(n^{-1/2})$, we see by (A4) that $\sigma_{n}^{2}$ is bounded by

$$\begin{aligned} \Biggl\Vert \operatorname{var}\Biggl( \sum_{t=1}^{n} \mathbf{ x}_{nt}\psi (e_{t})\Biggr) \Biggr\Vert =& \Biggl\Vert \sum_{t=1}^{n} ( \mathbf{x}_{nt})^{2} E\psi^{2} (e_{t}) \Biggr\Vert + 2 \Biggl\Vert \sum_{t=1}^{n-1} \sum_{j=t+1}^{n} \mathbf{x}_{nt} \mathbf{x}_{nj}^{\top} E\bigl(\psi(e_{t}) \psi(e_{j})\bigr) \Biggr\Vert \\ =& p \sigma^{2} + 2 \bigl\Vert \mathbf{S}_{n}^{-1} \bigr\Vert \bigl\Vert \mathbf {x}_{t}\mathbf{x}_{j}^{\top} \bigr\Vert \sum_{t=1}^{n-1} \sum _{j=t+1}^{n} \bigl\vert E\bigl(\psi(e_{t}) \psi(e_{j})\bigr) \bigr\vert \\ =& p \sigma^{2} + 2C \bigl\Vert \mathbf{x}_{t} \mathbf{x}_{j}^{\top } \bigr\Vert \\ =& p \sigma^{2}+2pC=O(1). \end{aligned}$$

In the view of $\mathbf{H}_{n}^{\top}\mathbf {H}_{n}=\mathbf {I}_{q}$ and Lemma 6,

42 $$ \sigma^{-1}\sum_{t=1}^{n} \mathbf{H}_{n}^{\top }\mathbf {x}_{nt} \psi(e_{t}) \xrightarrow{\mathfrak{D}}\mathrm {N}(\mathbf{0}, \mathbf{I}_{p}). $$

Thus Theorem 1 follows immediately from (41) and (42). □

### Proof of Theorem 2

Consider the model (11), without loss of generality, assume that the true parameter $\boldsymbol {\beta}(n)$ is equal to **0**. For any $\delta>0$, write

$$V_{n}=E \bigl\vert \psi\bigl(e_{1}+\delta d_{n}^{1/2}\bigr)-\psi\bigl(e_{1}-\delta d_{n}^{1/2}\bigr) \bigr\vert . $$

By the monotonicity of *ψ*, Schwarz’s inequality and (A2), we get, for sufficiently large *n*,

$$\begin{aligned}& EI \bigl( \bigl\Vert \hat{\boldsymbol {\beta}}(n) \bigr\Vert \leq\delta \bigr) \Biggl\vert \hat{\sigma}_{n}^{2}-n^{-1} \sum _{t=1}^{n}\psi^{2}(e_{t}) \Biggr\vert \\& \quad\leq V_{n}+2E \bigl\vert \psi(e_{1}) \bigl[\psi \bigl(e_{1}+\delta d_{n}^{1/2}\bigr)- \psi \bigl(e_{1}-\delta d_{n}^{1/2}\bigr) \bigr] \bigr\vert \\& \quad\leq V_{n}+2\delta V_{n}^{1/2}\rightarrow0. \end{aligned}$$

By Lemma 9,

$$\hat{\sigma}_{n}^{2}=n^{-1}\sum _{t=1}^{n}\bigl\{ \psi(e_{t}+h)- \psi(e_{t}-h)\bigr\} \xrightarrow{\mathbb{P}} E\psi^{2}(e_{1})= \sigma^{2}. $$

Consequently, (9) is proved.

As mentioned in Chen *et al.* [21], in order to prove (10), it is desired to prove that

$$(2nh)^{-1}\sum_{t=1}^{n}\bigl\{ \psi(e_{t}+h)-\psi(e_{t}-h)\bigr\} \xrightarrow{\mathbb{P}} \lambda. $$

Actually, by the monotonicity of $\psi(e_{t}+h)-\psi(e_{t}-h)$, and the assumption (8), applying Lemma 7 and Lemma 2, we get

$$\begin{aligned}& \operatorname{var} \Biggl\{ (2nh)^{-1}\sum _{t=1}^{n} \bigl[ \psi(e_{t}+h)- \psi(e_{t}-h) \bigr] \Biggr\} \\& \quad\leq \bigl(4n^{2}h^{2}\bigr)^{-1}E \Biggl[ \sum_{t=1}^{n} \bigl(\psi(e_{t}+h)- \psi(e_{t}-h)\bigr) \Biggr]^{2} \\& \quad\leq \bigl(4nh^{2}\bigr)^{-1}E\bigl[ \psi(e_{t}+h)-\psi(e_{t}-h)\bigr]^{2}\rightarrow0. \end{aligned}$$

On the other hand, since $\lim_{n\rightarrow\infty }[G(h)-G(-h)]/(2h)=\lambda$,

$$(2nh)^{-1}\sum_{t=1}^{n} \bigl[ \psi(e_{t}+h)-\psi(e_{t}-h) \bigr]=\bigl[G(h)-G(-h) \bigr]/(2h)+o_{P}(1) \rightarrow\lambda. $$

This completes the proof of Theorem 2. □

## Simulation

We evaluate the parameter estimates and the M-test for the powers by Monte Carlo techniques. Under the null hypothesis, the estimators of regression coefficients and redundancy parameters are derived by some M-methods such as LS method, LAD method and Huber method. Under the local alternative hypothesis, the powers of the M-test is obtained with the rejection region given by Theorem 1. In this section, the case of the NSD sequence is raised as follows:

$$X_{t}=a_{n}Y_{t}+b_{n}Z_{t}, \quad t=1,\ldots,n, $$

where $a_{n}$ and $b_{n}$ are positive sequences, $Y_{t}$ and $Z_{t}$ are negatively dependent (correspond to $\rho_{0} < 0$) random variables with the distribution

$$(Y,Z)\sim\mathrm{N}\bigl(\mu_{1},\mu_{2}, \sigma_{1}^{2},\sigma _{2}^{2}, \rho_{0}\bigr). $$

Now, we will prove that $(X_{1},X_{2},\ldots,X_{n})$ is a NSD sequence. Obviously, one may easily to check that

$$\operatorname{cov}(X_{t},X_{j}) < 0,\quad 1 \leq t< j \leq n. $$

As stated in Hu [10], the superaddictivity of *ϕ* is equivalent to $\partial^{2}\phi/ \partial x_{t}\partial x_{j} \geq0$, $1\leq t\neq j\leq n$, if the function *ϕ* has continuous second partial derivatives. In which, $\phi(x_{1},\ldots,x_{n})=\operatorname{exp}{(\sum_{t=1}^{n}X_{t})^{2}}$ can be chosen as a superadditive function. Note that the $\{X_{t}^{*}, 1 \leq t \leq n\}$ have same marginal distribution with $\{X_{t}, t=1,\ldots,n\}$ for each *t*, by Jensen’s inequality, the sequence $(X_{1},X_{2},\ldots,X_{n})$ is proved to be NSD since

$$\begin{aligned} \dfrac{E\phi (X_{1}^{*},X_{2}^{*},\ldots,X_{n}^{*} )}{ E\phi (X_{1},X_{2},\ldots,X_{n} )} =& E\operatorname{exp} \Biggl\{ \Biggl(\sum _{t=1}^{n}X_{t}^{*} \Biggr)^{2} -\Biggl(\sum_{t=1}^{n}X_{t} \Biggr)^{2} \Biggr\} \\ \geq& \operatorname{exp}E \Biggl\{ \Biggl(\sum_{t=1}^{n}X_{t}^{*} \Biggr)^{2} -\Biggl(\sum_{t=1}^{n}X_{t} \Biggr)^{2} \Biggr\} \geq1. \end{aligned}$$

Throughout the simulations, the Huber function is taken to be $\rho(x)={(x^{2}I(|x|\leq k))}/2+(k|x|-{k^{2}}/2)I(|x|>k)$, $k=1.345\sigma _{0}$. The explanatory variables are generated from two random models and all of the simulations are run for 1,000 replicates and calculate the averages of the derived estimates to avoid the randomness impact.

The linear model with NSD errors is given by $y_{t}=\beta_{0}+\beta _{1}x_{t}+e_{t}$, $e_{t}=Y_{t}+Z_{t}$, $t=1,2,\ldots,n$, where the NSD errors $\{e_{t},t=1,2,\ldots,n\}$ are assumed to follow a multivariate mixture of normal distribution with joint distribution $(Y,Z)\sim\mathrm{N}(\mu _{1},\mu _{2},\sigma_{1}^{2},\sigma_{2}^{2},\rho_{0})$, $\rho_{0}<0$. The null hypothesis is $H_{0}:(\beta_{0},\beta_{1})^{\top}=(1,2)^{\top}$. The sample size is taken to be $n=100$, $n=500$, $n=1{,}000$. The joint distribution is taken to be $(Y,Z)\sim\mathrm{N}(0,0,1,16,-0.5)$. The explanatory variables $x_{t}$ are generated by the following two random models: I. $x_{t}=5\mathrm{u}_{t}$, $1\leq t \leq n$; II. $x_{t}=\sin (2t)+1.5\mathrm{u}_{t}$, $1\leq t \leq n$, where u obeys a standard uniform distribution $\mathrm{U}(0,1)$.

**Figure 1:**
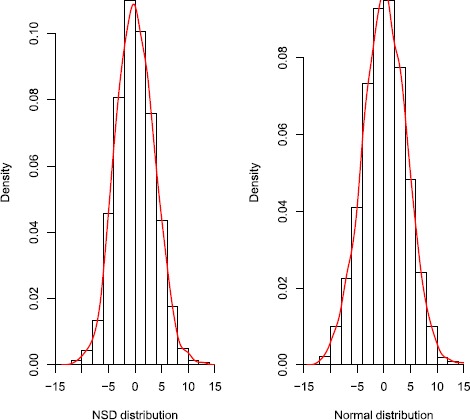
**Histograms and fitted distributions of M-estimates residuals with different explanatory variables and M-methods (sample size is** $\pmb{n=1{,}000}$ **).**

Firstly, we generate a NSD sequence by the Gibbs sampling technique. Figure 1 shows the fitted distribution (full line) of NSD is close to the normal distribution, relatively speaking, the NSD distribution tends to behave a truncated distribution feature.

**Figure 2:**
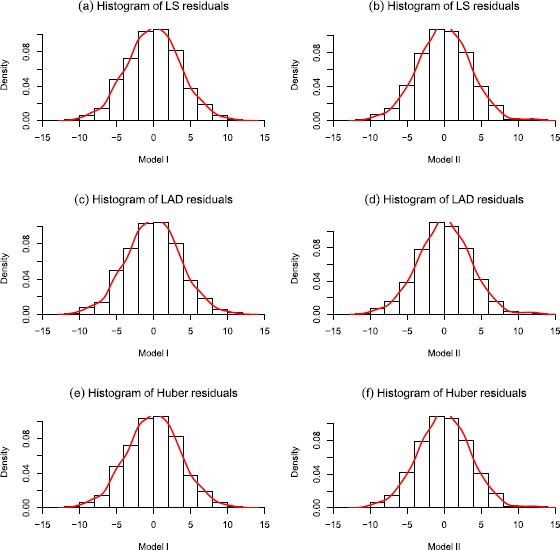
**Histograms and fitted distributions of M-estimates residuals with different explanatory variables and M-methods (sample size is** $\pmb{n=1{,}000}$ **).**

**Figure 3:**
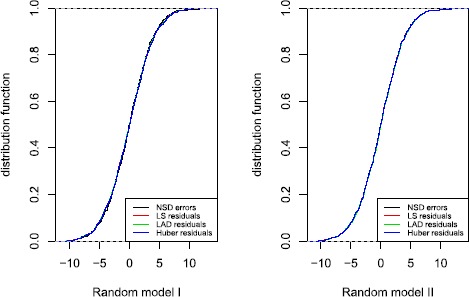
**A comparison fitted distribution functions of residuals and assumed NSD errors (sample size is** $\pmb{n=1{,}000}$ **).**

Next, we evaluate the estimators of regression coefficients and redundancy parameters under the null hypothesis, Table 1 illustrates that the M-methods are valid (the corresponded M-estimates are close to true parameters $\beta_{0}=1$, $\beta_{1}=2$) and the estimators of redundancy parameters are effective (one may easily to check that $\sigma ^{2}=13$ and $\lambda=1$ when the convex function is taken to LS function, for other estimates, although their values are different based on different methods, the sign and significance remain the same, so the general conclusions remain the same). Additionally, with the increasing sample size, the estimations are more and more accurate. In fact, the estimations behave well though the sample size is not large ($n=100$). As excepted, the fitted residual densities are close to the assumed NSD errors in Figure 2, and all of them still show a truncated distribution feature. Figure 3 checks the residuals are NSD by using the empirical distribution to approximate the distribution function, which supports the NSD errors assumption.

**Table 1:** **The evaluations of regression coefficients and redundancy parameters**

**Estimates**	***n***	**LS**		**LAD**		**Huber**	
		**I**	**II**	**I**	**II**	**I**	**II**
$\hat{\beta}_{0}$	100	1.031	0.985	1.016	0.978	1.006	1.007
	500	0.994	0.994	1.008	1.006	1.003	1.006
	1000	1.002	0.999	1.000	1.006	1.002	1.003
$\hat{\beta}_{1}$	100	1.983	2.008	1.992	2.016	2.131	2.131
	500	2.003	2.011	1.997	2.003	1.996	1.992
	1000	1.997	1.997	1.999	1.998	1.996	1.994
$\hat{\sigma}^{2}_{n}$	100	12.764	12.671	0.984	0.987	9.095	9.100
	500	12.965	12.941	0.997	0.997	9.193	9.206
	1000	12.967	12.956	0.998	0.998	9.208	9.291
$\hat{\lambda}_{n}$	100	1.000	1.000	0.282	0.282	0.825	0.825
	500	1.000	1.000	0.241	0.241	0.822	0.823
	1000	1.000	1.000	0.233	0.234	0.822	0.821

**Figure 4:**
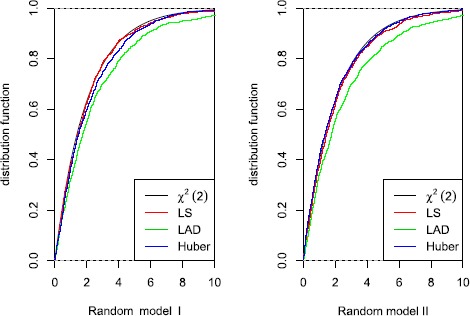
**A comparison fitted distribution functions of** $\pmb{2\hat{\lambda}_{n}\hat {\sigma}_{n}^{2}M_{n}}$ **and the central chi-squared distribution with two degrees (sample size is** $\pmb{n=1{,}000}$ **).**

Finally, we study the empirical significant levels and the powers of M-test. Under the local hypothesis, $2\hat{\lambda}_{n}\hat{\sigma }_{n}^{2}M_{n}$ has an asymptotic central chi-squared distribution with two degrees of freedom by Theorem 1 and Theorem 2, we may reject the null hypothesis if the simulative value $2\hat{\lambda}_{n}\hat {\sigma }_{n}^{2}M_{n}\in W$ in (7). Table 2 presents the powers at significance levels $\alpha=0.05$ and $\alpha=0.01$ for various choices of M-methods, explanatory variables and different sample sizes $n=100$, $n=500$, $n=1{,}000$. The result represents that the empirical significant levels are close to the nominal levels, consequently, the M-test is valid. Figure 4 illustrates that $2\hat{\lambda}_{n}\hat {\sigma}_{n}^{2}M_{n}$ can approximate the central $\chi_{2}^{2}$ well by comparing the empirical distributions of $2\hat{\lambda}_{n}\hat {\sigma}_{n}^{2}M_{n}$ with $\chi_{2}^{2}$, which implies that the M-test is valid under the local alternatives.

**Table 2:** **The powers of the M-test with NSD errors, ‘∗’ is for the nominal significant levels**

***n***	**Significance levels**	**LS**		**LAD**		**Huber**	
		**I**	**II**	**I**	**II**	**I**	**II**
100	0.05^∗^	0.063	0.068	0.082	0.079	0.062	0.062
	0.01^∗^	0.013	0.011	0.028	0.019	0.013	0.016
500	0.05^∗^	0.059	0.057	0.064	0.052	0.054	0.059
	0.01^∗^	0.009	0.013	0.020	0.013	0.009	0.012
1000	0.05^∗^	0.056	0.056	0.062	0.052	0.048	0.057
	0.01^∗^	0.012	0.015	0.013	0.011	0.010	0.013

## Conclusions

The results presented here generalize conclusions in [20–22]. In the simulations it turns out that the M-tests for the linear model with NSD errors are insensitive to different choices of M-methods and explanatory variables, therefore it shows robustness, which illustrates that the M-test is effective.
